# Selections and Abstracts

**Published:** 1915-05

**Authors:** 


### SELECTIONS AND ABSTRACTS

### ULCER AND CANCER OF THE STOMACH. THE CAMPAIGN AGAINST CANCER.

### SYMPOSIUM: LOOKING TO THE INCREASED KNOWLEDGE OF CANCER.

### THE PRESENT STATUS OF CANCER RESEARCH.

BY LEO LOEB, M. D., PHILADELPHIA.

(Continued from Last Number.)

### CANCER OF THE MOUTH AND LIP.

BY ERNEST LAPLACE, M. D., PHILADELPHIA.

Cancer of the mouth and lip is especially destructive of life, causing eleven per cent of all deaths from cancer, and being about ten times more frequent in men than in women. Two elements, at least, enter as explanatory factors: (1) Free metastases, owing to the vast lymphatic supply of the mouth and tongue by which these vessels reach the lymphatic ganglia of the neck; (2) the excessive virulence soon acquired by malignant disease of the mouth and tongue, owing to the added irritation of the saliva and bacteria of the mouth, and also the mechanical irritation of food.

According to Sappey, lymphatics from the floor of the mouth pass through the mylohyoid muscle into the submaxillary glands and partly into the deep cervical chain. The lymphatics of the deep surface of the cheek and roof of the mouth join the internal maxillary glands. The lymphatics of the tongue run back toward the ranine vein, and, after passing through several small ligual glands on the hyoglossus of the front part of the tongue, pass with the lymphatics of the floor of the mouth through the mylohyoidean muscles into the submaxillary glands.

The cancer may trespass its fibrous bounds, and, finding a lymphatic vessel open, penetrate into the caliber. Then the case is beyond the domain of a surgical cure, as the cancer cells have traveled quite a distance through one of the avenues above described. This metastasis need not necessarily start to grow immediately and concomitantly with the original growth, but may be held in abeyance, hibernating, as it were, and giving no appreciable sign of its presence for a long time. A metastatic enlarged gland or infiltrating growth may develop later on or may start only when the original growth has been removed.

A second cause of the great virulence of cancer of the mouth is that as soon as the cancerous proliferation begins, the many virulent germs of the mouth develop secondary infections, which add their irritation to the cancerous process and accelerate its growth, increasing its infiltration and metastasis. The noli me tangere of the past forbade mechanical irritation to skin epitheliomata. In the mouth is found the constant mechanical irritation of food or of a carious tooth. The secretions of the mouth and its virulent germs are a constant source of chemical irritation to the epithelioma of the mucous membrane of the mouth and tongue.

Cancer does not always start with the same degree of virulence, any more than the streptococcic infection does. As there may be an acute, subacute or chronic streptococci infection, creating clinical pictures, so familiar to us all, so also with cancer this is relatively true. Epitheliomata of the mouth may develop more or less rapidly. An acute or virulent streptococcic infection finds the lymphatics open and the lymphatic infection spreads rapidly; sometimes a hemic infection also results. A subacute or chronic infection develops slowly enough to allow the lymphatic to close, and subsequently the formation of fibrous tissue which retains the infection within bounds. Cancerous infection of the mouth and tongue presents some analogy to this. We find small, warty or papillomatous growths existing for an indefinite period on the lip or on the floor of the mouth, without much tendency to spread; no infiltration or spreading takes place because the mild or chronic nature of the infection has allowed the formation of fibrous tissue to protect the subcutaneous cellular tissue.

Should a rapid development of tissue take place or should constant irritation exist, the newly forming cells will be so plentiful as not only to accumulate on the surface and produce a tumor of greater size, that is, an ordinary epithelioma, but also to grow in the depth of the tumor, gradually perforating the isolating basement membrane entering the lymphatics, infiltrating the cellular tissue and creating a condition beyond the certainty of a complete possible removal; that is, a genuine carcinoma or cancer. This rapid development and infiltration may sometimes exist from the first, owing to the peculiar virulence of the cancer, and may establish the acute or fulminating condition of malignancy, as in melanotic cancer.

This, therefore, is the crucial point in the diagnosis whereupon the surgical treatment should be based. Have any cancerous cells perforated the basement membrane of the skin of the lip or mucous membrane of the tongue or mouth? If not, a local operation is all that may be required, removing the growth and a moderate amount of surrounding tissue. If infiltration has started, we must concede that theoretically as well as from practical experience no operation could be too extensive, if our purpose is, as it should be, the removal not only of the disease but of such cells as might have migrated to a distance; this includes the deeper cervical ganglia of the neck, even when these show clinically no perceptible sign of infection, for, as above stated, the cancer cell may be nestling there, in abeyance, to start its own growth, perhaps only after the original disease has been removed, thus explaining the recurrence of the disease.

There may be a suspected precancerous stage in a patient. It may be detected from (1) Heredity; (2) local appearance of the skin surface or mucous membrane; (3) hemolytic test. While modern tendency is to discredit heredity somewhat as an etiological factor in disease, the appearance of cancer is still more frequent in families where there is a cancerous history. Locally there is a peculiar, glossy condition of the surface of the skin showing a thin layer of epithelioma through which the capillaries appear rather prominently. There is a tendency to scabs forming here and there, and persisting, showing a slight infection of the papillary layer of the skin, or areas of leukaplasia or excoriated patches in the oral cavity.

Kelling has drawn our attention to the hemolytic property of the blood serum of cancerous patients, and Crile's researches, based upon Kelling's, have further developed a method which may eventually prove of scientific precision for the earliest possible diagnosis of a cancerous condition, or even a precancerous stage.

Clinically we must concede that epithelioma seems to be at first a strictly local infection of the skin or mucous membrane. A long-continued irritation predisposes a particular spot to the infection. Moles, warts, angiomas and especially pigmented warts are prone to develop the infection; hence, these should be promptly removed before their malignant infection. Elderly people, careless of facial hygiene, are subject to the hypertrophies of the skin. The added mechanical irritation produced by picking the spot with the fingers or the use of the pipe or tobacco smoke aggravates the infection. Carious teeth, accompanied by improper mouth hygiene, furnish a localizing cause.

The early, differential diagnosis between syphilitic, cancerous and tuberculous ulcerations of the lip and mouth is most important. The classical, clinical, differential elements as described in our text-books, are, to say the least, insufficient. We are all aware of the deceptive leukoplasia of the mouth, which, though generally syphilitic in origin, is unfortunately very often the fundamental base of a future epithelioma, which would be too extensive for complete removal should we wait for vast infiltration and glandular metastasis to seal the diagnosis. Clinical diagnosis has played a more decisive part in the surgical past than it will in the surgical future. The day of more precise and scientific early diagnosis has come, based on laboratory methods, which give results that are absolutely specific for tuberculosis, syphilis and cancer. The tentative mercurial treatment, while useful, has been abused in the past. It should not be persistently continued when no evident improvement takes place for fear of aggravating the condition.

The Pirquet cutaneous tuberculous reaction or the Calmette conjunctival reaction will determine tuberculosis, also

Rosenberg's method of discovering tubercle bacilli in the blood. The Wasserman hemolytic reaction will determine syphilis, and the Kelling test as modified by Crile may throw much light upon the cancerous nature of ulceration or the precancerous stage of the patient. An early diagnosis is our sheet anchor and these labortory tests are the key to the problem, or at least will greatly help to determine the positive diagnosis, when clinical signs are not definite, or are insufficient. Of course, we hope that some day, the earliest possible diagnosis being made, a serum will be used that will cause the absorption and disappearance of the incipient, cancerous ulcer and protect the system against future developments of the disease, as diphtheria antitoxin dissolves sclerotic croupous membrane of the suffering child. May this blessing come with the least delay. In the meanwhile surgery is the only remedy and this, provided it can completely eradicate the disease.

I have shown above why mouth epithelioma is from the first especially virulent, and how it is most prone to metastasis. For surgery to achieve success, it must actually do what it pretends to do; that is, remove the disease and sufficient surrounding tissue to ensure the exeresis of migrating cells if any; better still, remove the epithelioma before it has become a cancer; that is, before the limiting basement membrane has been perforated by the infiltrating cancer cells, allowing them to penetrate the lymphatics. This is best done with the knife. The growth is removed, the lymphatics are necessarily left opened. For fear that any malignant cells might have fallen into the wound exposing the patient to a local recurrence by reinfection, the wound should be thoroughly cauterized with the thermocautery, thus effectually destroying all the surface cells and sealing the opened lymphatic vessels. This treatment should also be practiced for epithelioma of the lip; a copious removal of the growth, cauterization and, later, suturing of the parts. I must here decry the use of all cancer pastes, which sometimes successfully cause a slough of the growth and of the surrounding tissue if the growth be small, but often only irritate the deeper cells of the growth, causing them to develop with redoubled activity and starting the infiltration of cancerous cells, which will finally elude all treatment.

The intent and purpose of this symposium is to depart from the older and known ineffectual methods, whereby vast operations are performed. When these are indicated there has not been an early diagnosis and it is too late for a successful elimination of all cancer cells. These operations, no matter how vast and by whom performed, may be classed as surgical feats, with absolutely futile and discouraging results. They must be relegated to an historical past, as amputations for hospital gangrene have been. They must be supplanted by the early and successful operation as the causes of hospital gangrene are today, nipped in the bud.

An early clinical and laboratory diagnosis, the knife and thermocautery constitute the surgeons' course. The use of x-rays and radium is of doubtful value to a growth in the mouth. I would advise its use only as an extra postoperative precaution for prophylaxis. Its action is much more encouraging in epithelioma of the face and skin generally.

Successful surgery, however, does not claim to alter the cancerous diathesis. This remains the same after, as it was before, the surgical operation. Hence in two of my successful cases of cancer of the mouth and of the tongue, I was confronted with a recurrence three and four years afterwards of carcinoma of the rectum and carcinoma of the liver. This confirms a growing belief that cancer is due more to intrinsic than extrinsic causes. If this were not the case, everyone could be inoculated with the disease, whereas we know that cancer can not be transplanted to another human subject, but is only re-inoculable on the patient himself.

CONCLUSIONS.

1. Cancer of the mouth shows itself amenable to successful surgical treatment only in the earliest stages.

2. A diagnosis by laboratory methods based upon the intrinsic changes of the body alone can furnish the diagnosis sufficiently early.

3. This should be combined with the clinical diagnosis.

4. An immediate and copious removal of the growth should be practiced, followed by thorough thermocautery cauteridation.

5. We should remember that until a method of immunizing the patient is discovered, whereby the intrinsic predisposing condition of the patient is changed to a normal condition, no surgical operation nor any method of treatment will insure a non-recurrence of the disease either in situ or in some other part of the body.

6. The excision of a bit of tissue for diagnostic purposes must be deprecated, for fear of infecting the deeper tissues.

7. The profession at large should abstain from the usual cauterizations, used as a tentative treatment which may aggravate the case to the extent of placing it beyond a surgical cure. Every wart, mole or ulcer in the mouth, no matter how benign in appearance, is a potential cancer when the patient possesses those intrinsic causes which give susceptibility to malignancy.

---

### THE EARLY DIAGNOSIS AND BEST TREATMENT OF CANCER OF THE RECTUM.

By Robert W. Stewart, M. D., Surgeon to Mercy Hospital, Pittsburg.

The frequency of cancer of the rectum, the intolerable suffering it, if unrelieved, entails, together with its amenability to partial or complete relief by operative interference, gives this disease a prominent place among the surgical maladies.

The onset of cancer of the rectum is usually insidious, the first symptom often being a sense of discomfort that is relieved by the evacuation of the bowels. If the growth is situated high up in the rectum, this discomfort may not be present until the disease has made considerable progress. Sometimes the disease gives its first manifestation by an acute obstruction of the bowels, either by a blocking of the lumen by a fecal concretion, or a prolapse of the bowel immediately above the obstruction.

If the growth is within reach of the finger, the diagnosis is easy, even in the early stages of the disease. Nevertheless, errors in diagnosis have been made by most competent operators, and much discredit has more than once been thrown upon the profession by the cure, by quack methods, of cases of alleged cancer, the patient having previously refused to submit to an operation.

Acute inflammatory conditions producing perirectal infiltration, tubercular and syphilitic disease or benign neoplasms are the conditions usually mistaken for cancer of the rectum, and it is probable that the cures following colostomy have really been for one of the above conditions. Mayo Robson, in a recent article, states: "No less than five times in the last twelve years I have had patients sent to me suffering from supposed cancer of the rectum or sigmoid flexure with obstructive symptoms necessitating a colotomy, in whom, after periods varying from one to three years, I have been called on to close the artificial anus because of the complete disappearance of the supposed growth."

Cancer of the rectum is not necessarily a disease of middle or advanced life; it may occur in early adult life, one of my cases producing intestinal obstruction and death at the age of seven.

A point of very great importance in the operative treatment of cancer of the rectum is the slowness with which the growth tends to involve the surrounding structures, or spread by lymphatic extension. The inference from this is obvious, that here, as in cancer of the breast, early radical excision, extending well into apparently healthy tissue, offers a very fair promise of a cure of the disease.

Cripps, from an extensive experience, estimates that in only about thirty per cent of the cases coming to the surgeon are the cancers in a suitable condition for excision, the other seventy per cent of the patients having lost their chance of a cure by applying too late to justify radical interference.

In considering the question of treatment, it is not always easy to determine which cases should be subjected to excision. In few surgical conditions does so much depend on the judgment of the surgeon. From the patient's standpoint, good surgical judgment is of far more importance than great operative skill, as he may be injured by an excision, no matter how skillfully performed, if the growth has clearly extended beyond operative limits, as his suffering would have been as effectively relieved by a simple colostomy.

Provided the patient's general condition will permit of a major operation, the carcinomatous growth should be excised in those cases where the bowel is movable, indicating the absence of perirectal cancerous infiltration. It is especially important, although not absolutely essential, that the rectum be free from anterior adhesions.

In one case in which I found it necessary to excise the posterior wall of the vagina, the patient remained well for four years, dying from typhoid, without recurrence of the cancer. In another case, I removed the rectum, uterus and greater portion of the vagina which were involved in the cancerous growth; the patient still remains free from recurrence, after an interval of three and one-half years. However, if the prostate or bladder is involved, an excision is contraindicated, and probably would be worse than useless, as it would facilitate the dissemination of the disease.

Fixation of the rectum posteriorly is of less serious importance, as in this direction we can dissect with great freedom, although extensive posterior involvement usually indicates hopeless lymphatic invasion. While we are unanimous in advising excision wide of the growth in suitable cases, there is still a great difference of opinion as to the best method of performing the operation. The writer does not venture to speak authoritatively on this subject, but only gives his opinion for what it is worth.

If the growth is situated within the lower third of the rectum, and does not progress so far as to indicate its extension to the lymphatics, it may be excised by the perineal method, removing if necessary, the coccyx and posterior wall of the vagina and suturing the proximal end of the bowel to the margin of the wound. If the growth is situated in the middle third of the rectum, it may be excised in a similar manner, with the addition, however, of a more extensive bony excision and usually opening the peritoneal cavity. This operation, named after Kraske, is open to the objection that, after a great deal of dissection has been done, it will frequently be discovered that the disease has extended so far along the lymphatics, or there is so much intrapelvic involvement, that a cure is out of the question. In order to save the sphincters, the diseased segment of bowel has frequently been excised, suturing the proximal and distal ends so as to insure subsequent continence. This may be described as the ideal operation, as its object is to restore the act of defecation to a normal condition, but, unfortunately, the ideal is difficult and often impossible of attainment. This operation rarely permits of a sufficiently wide dissection to give a reasonable hope of nonrecurrence of the disease. A cicatricial stricture may develop at the point of union of the upper and lower segment and, should there be a local recurrence of the disease, the function of the bowel will again be interfered with, necessitating in all probability a subsequent colostomy. It is also difficult to prevent the stitches from giving way, and there is a great likelihood of perirectal abscesses and fistulous communications which subject the patient to great risks of death from sepsis.

If performed at all, this operation should be limited to those cases of cancer where the growth is clearly confined to the bowel.

In the great majority of cases, the operation to be recommended for the removal of the cancerous growth is the combined abdominal and perineal methods, by which the entire rectum is excised, including the lymphatic-bearing area lying posterior to the rectum.

I will avoid the details of the preparation of the patient for this operation, some of which, such as the thorough evacuation of the bowels, are of the utmost importance. The patient is placed in the Trendelenberg position, and the abdomen opened by a median incision immediately above the pubes. The intestines are packed out of the way and the pelvic cavity carefully examined, to determine the extent of the growth. Sometimes the examination will show an unexpected invasion of the lymphatics or pelvic organs, precluding the possibility of a cure by any operation, and in such cases an attempt at excision should be abandoned, and a colostomy substituted. If the conditions are favorable for an excision, the sigmoid flexure is drawn into the wound in such a way as to render its lower portion taut, and bring the upper portion of the rectum well into view. The peritoneum is then incised by parallel incisions extending on either side of the bowel from the lower third of the sigmoid to the point where the peritoneum is reflected anteriorly to the vagina or bladder as the case may be. A blunt dissection now isolates the lower portion of the sigmoid from its posterior connections, care being taken to tie off the arterial supply, and to avoid the ureters. The dissection is carried downwards as far as possible, detaching the upper portion of the rectum and the lymphatic-bearing tissue posterior to the gut. We now have the lower third of the sigmoid and at least the upper half of the rectum freed from their peritoneal and vascular connections. The sigmoid should be drawn well out of the wound and the pelvic cavity packed with warm moist sponges; the sigmoid is next divided between two clamps at about the junction of its middle and lower third, and the cut ends invaginated by purse-string sutures, the ends of the sutures being left long and attached to the two clamps. The next step is to place the patient in the lithotomy position and complete the removal of the bowel by a perineal dissection. It may be necessary to remove the coccyx in order to obtain sufficient room for easy dissection. The rectum should be carefully detached from its anterior connections, especially when the prostate and seminal vesicles are exposed, or the posterior wall of the vagina may be removed if considered advisable; the dissection should be carried forward anteriorly until the pelvic cavity has been opened at the lowest point of the intra-pelvic dissection. Through this opening an assistant passes the blades of the forceps, carrying the purse-string suture on the distal portion of the bowel. This is drawn down into the perineal wound and, while traction is made on it, the bowel is rapidly detached from its posterior sacral connections, keeping wide of the bowel and close to the sacrum, in order to remove the lymphatic tissue. If this portion of the operation is carried out rapidly, the hemorrhage may be ignored until the rectum is removed. After the hemorrhage is controlled, the walls of the cavity are approximated by buried sutures, making provision, however, for free drainage. The patient should now be placed in the ordinary position; the gloves and field clothes changed, and the end of the sigmoid brought through a gridiron incision on the left side about two inches above Poupart's ligament. The bowel is then slipped under the skin, brought to the surface and sutured to a small skin incision over Poupart's ligament, after Bailey's method. This leaves about two inches of the bowel lying subcutaneous at a point where it can be compressed by a truss. The intrapelvic sponges are now removed, and the pelvic toilet completed by suturing the peritoneal edges together. The abdominal wound is closed without drainage, as is also the small skin incision over the muscle-splitting wound. After the dressings are applied to the abdominal wound, the purse-string suture closing the end of the sigmoid should be removed, and the inverted end of the bowel everted, separate dressings applied, which should be changed as soon as soiled. If considered advisable, the removal of the purse-string suture may be deferred until the following day.

It is scarcely necessary to say that this is an operation which should not be undertaken except by the experienced abdominal surgeon. The rectal specialist who undertakes this work, without previous experience in abdominal surgery, should, for his own peace of mind, be born with an easy conscience.

Total excision of the rectum will always be an operation of considerable gravity and associated with a definite mortality, and should not be undertaken except in those cases which, after careful rectal and intrapelvic examination, give at least a reasonable hope of a permanent recovery.

However, if we consider the comparatively late extension of the cancer beyond the limits of the rectum, it ought, in suitable cases, to give at least as good prospects of permanent recovery as does the radical removal of cancer of the breast.

It is difficult to give reliable statistics of the mortality of this operation, and still more difficult to get the percentage of permanent cures. Reports of individual cases should be ignored and I am not yet in a position to give immediate or final results based on the reports of the large surgical clinics.

While excision of the rectum is the operation of choice and is the only one which offers the hope of a permanent cure, it is, unfortunately, only applicable in the minority of cases in which the patients come to the surgeon for relief. Nevertheless, much may be done in the worse cases, not only to render the patient's condition more comfortable, but also to prolong his life. I refer to the operation of inguinal colostomy, the steps of which are too well known to require description. The principal objection to colostomy is the incontinence that follows; but this objection, which is also equally applicable to total excision, carries but little weight, when we consider that the patients, suffering from carcinoma of the rectum, sooner or later lose control of the bowel, also that in the majority of cases of colostomy a degree of control is regained that is sometimes surprising, many patients remaining useful members of society. On the other hand, the operation is exceedingly simple and should have but a slight mortality. It gives immediate relief to the rectal tenesmus and it has been noted by nearly every operator of experience that as a result of the arrest of the fecal current, and the consequent cessation of a direct irritation of the ulcerating surface, the cancerous growth not infrequently becomes less active, and even quiescent, and the patient's life materially prolonged. Cripps mentions one patient who lived eight years after colostomy for cancer.

The necessary bevity of this paper prevents me from discussing the nonoperative treatment of cancer of the rectum, or the consideration of operative methods that are now obsolete.

(To be continued.)

